# Molecular Phylogeny and Dating of Forsythieae (Oleaceae) Provide Insight into the Miocene History of Eurasian Temperate Shrubs

**DOI:** 10.3389/fpls.2018.00099

**Published:** 2018-02-05

**Authors:** Young-Ho Ha, Changkyun Kim, Kyung Choi, Joo-Hwan Kim

**Affiliations:** ^1^Department of Life Science, Gachon University, Seongnam, South Korea; ^2^Korea National Arboretum, Pocheon, South Korea

**Keywords:** *Abeliophyllum*, biogeographic origin, chloroplast genome, *Forsythia*, Forsythieae, molecular dating, phylogenetic relationship

## Abstract

Tribe Forsythieae (Oleaceae), containing two genera (*Abeliophyllum* and *Forsythia*) and 13 species, is economically important plants used as ornamentals and in traditional medicine. This tribe species occur primarily in mountainous regions of Eurasia with the highest species diversity in East Asia. Here, we examine 11 complete chloroplast genome and nuclear *cycloidea2* (*cyc2*) DNA sequences of 10 *Forsythia* species and *Abeliophyllum distichum* using Illumina platform to provide the phylogeny and biogeographic history of the tribe. The chloroplast genomes of the 11 Forsythieae species are highly conserved, except for a deletion of about 400 bp in the *accD*–*psaI* region detected only in *Abeliophyllum*. Within Forsythieae species, analysis of repetitive sequences revealed a total of 51 repeats comprising 26 forward repeats, 22 palindromic repeats, and 3 reverse repeats. Of those, 19 repeats were common and 32 were unique to one or more Forsythieae species. Our phylogenetic analyses supported the monophyly of *Forsythia* and its sister group is *Abeliophyllum* using the concatenated dataset of 78 chloroplast genes. Within *Forsythia*, *Forsythia likiangensis* and *F. giraldiana* were basal lineages followed by *F. europaea*; the three species are characterized by minutely serrate or entire leaf margins. The remaining species, which are distributed in East Asia, formed two major clades. One clade included *F. ovata*, *F. velutina*, and *F. japonica*; they are morphologically supported by broadly ovate leaves. Another clade of *F. suspensa*, *F. saxatilis*, *F. viridissima*, and *F. koreana* characterized by lanceolate leaves (except *F. suspensa* which have broad ovate leaves). Although *cyc2* phylogeny is largely congruent to chloroplast genome phylogeny, we find the discordance between two phylogenies in the position of *F. ovata* suggesting that introgression of the chloroplast genome from one species into the nuclear background of another by interspecific hybridization in East Asian *Forsythia* species. Molecular dating and biogeographic reconstructions suggest an origin of the Forsythieae species in East China in the Miocene. Distribution patterns in *Forsythia* indicated that the species were radially differentiated from East China, and the speciation of the European *F. europaea* was the result of both vicariance and dispersal in the late Miocene to Pliocene.

## Introduction

Forsythieae H. Taylor ex L. Johnson (Oleaceae) is comprised of two genera, *Forsythia* Vahl and *Abeliophyllum* Nakai (Angiosperm Phylogeny Group [APG], 2016). *Abeliophyllum* is a monotypic and endemic genus in Korea characterized by samara-type fruits and white flowers (Kim, 2007). *Forsythia* consists of 13 locally derived endemic species restricted to certain geographical regions. They are deciduous shrubs characterized by opposite simple or rarely 3-parted to 3-foliolate petiolate leaves, and yellow flowers blooming in the early spring before leaves (Chang et al., 1996; Wu et al., 2010). Nowadays, many ornamental species of *Forsythia* have been created by horticultural scholars to improve their cold hardiness and enrich the colorful display of flowers (DeWolf and Hebb, 1971). Additionally, the fruits of *Forsythia suspensa* Vahl and *F. viridissima* Lindl. have been used in traditional herbal medicine in Korea and China (Xia et al., 2009; Ryuk et al., 2010). In light of the need to preserve native species and utilize their genetic information in various phylogenetic and conservational studies, we determined the chloroplast (cp) genome sequences of Forsythieae species.

Since Nakai (1919b) described *Abeliophyllum* as a new genus, taxonomical position of the genus within the Oleaceae was contentious and many studies have been performed to resolve their relationships (O’Mara, 1930; Sax and Abbe, 1932; Taylor, 1945; Johnson, 1957; Maekawa, 1962; Lee and Park, 1982a,b; Lim et al., 1989). Based on morphological and anatomical characteristics of the fruit (winged samara) and pollen aperture type (tricolporate), *Abeliophyllum* is most closely related to *Fontanesia* (Taylor, 1945; Johnson, 1957). In contrast, *Abeliophyllum* is most closely related to *Forsythia* according to the same chromosome number (*x* = 14) and karyotype pattern (Sax and Abbe, 1932; Tae et al., 2005). While recent molecular phylogenetic studies have verified *Forsythia* and *Abeliophyllum* are sister groups in Oleaceae (Lee et al., 2007; Kim and Kim, 2011), the relationships within *Forsythia* remain controversial. Using restriction fragment length polymorphism, Kim (1999) suggested that *Forsythia* species are divided into four groups according to their geographical distribution. Lee (2011), however, proposed the division of *Forsythia* species into two groups, the *F. koreana* complex and *F. nakaii* complex, based on morphological characters such as petiole length, darkness of the petals, floral tube and lobes, and sepals. In contrast, Kim and Kim (2011) proposed three *Forsythia* lineages using internal transcribed spacers (ITS) and plastid DNA *trnL-F* and *matK* gene sequences. Previous studies resolved different relationships (especially between *F. suspensa*, *F. saxatilis* Nakai, and *F. viridissima*) and did not fully resolve the complexes/clades because of insufficient supporting characters (Kim, 1999; Kim and Kim, 2011). These incongruous relationships reveal difficulties in recognizing morphologically ambiguous species.

In North Hemisphere, disjunctive pattern of plants has been intensively studied between East Asia and North America (Wen, 1999; Donoghue and Smith, 2004) since Asa Gray (Gray, 1846) first reported the phenomenon. In contrast, disjunction of species between East Asia and Europe has been less focused because the migration of plants between two regions could be due to continuous mountain chains toward east–west direction across Eurasia (Green, 1972b). *Forsythia* species are distributed in Eurasia, with high species diversity in East Asia—six species are distributed in China (*F. giraldiana* Lingelsh., *F. likiangensis* Ching & Feng ex P.Y.Pai, *F. mandschurica* Uyeki*, F. mira* M.C.Chang, *F. suspensa*, and *F. viridissima*), four in Korea (*F. koreana* Nakai, *F. ovata* Nakai, *F. saxatilis*, and *F. velutina* Nakai), and two in Japan (*F. japonica* Makino and *F. togashii* Hara) (Yamazaki and Noshiro, 1993; Chang et al., 1996; Lee, 2002). *F. europaea* Degen & Bald is disjunctively distributed in northern Albania and adjacent parts of the former Yugoslavia (Green, 1972a; Willis and Shaw, 1973; Mabberley, 1997). Although the distribution of *Forsythia* species shows a noticeably distinct pattern between East Asia and Europe, the biogeographical study by Kim (1999) is the only study that examined this pattern based on chloroplast (cp) DNA substitution rate. Additionally, age estimation of the Oleaceae was conducted at higher taxonomic level with only a few species representing *Forsythia* (Wikström et al., 2001; Bell et al., 2010). Therefore, biogeographic origin of the tribe Forsythieae and their biogeographic patterns of distribution between East Asia and Europe has been not addressed.

The cp genome, containing genes coding for photosynthesis, is generally maternally inherited in Angiosperm (Corriveau and Coleman, 1988). It has a conserved quadripartite structure consisted of two copies of inverted repeats (IRs), a large single copy (LSC) region, and a small single copy (SSC) region (Palmer, 1991; Raubeson and Jansen, 2005). In addition, the analyses using whole cp genome sequences were efficient and essential for verifying variations in phylogeny as a result of sequence divergence among plant species at genus and tribal levels (Huang et al., 2014; Feng et al., 2016; Givnish et al., 2016). Due to variations in the cp genome, more studies have focused on genomic events such as simple sequence repeats (SSRs) (Park et al., 2016), indels (Ye et al., 2014), expansions/contractions (Yang et al., 2013), and inversions (Lee et al., 2007; Kim et al., 2016). Moreover, complete cp genome sequences have been used extensively to resolve phylogenetic relationships (Moore et al., 2007), evaluate species identification (Wu et al., 2010; Nock et al., 2011), and reveal biogeographical history (Givnish et al., 2016).

Next-generation sequencing (NGS) technologies provide a cost-effective method by accessing extensive amounts of data that can provide insights into the phylogenetic relationship and biogeographic history of plants (Hörandl and Appelhans, 2015). This technology can be used to recover whole cp genomes, mitochondrial data, and numerous nuclear markers which can aid in resolving Forsythieae species. Here, we obtained complete cp genome sequences and nuclear single-copy gene, *cycloidea2* (*cyc2*) that encodes a transcription factor involved in the evolution of corolla zygomorphy (Zhong and Kellogg, 2015) of *Abeliophyllum distichum* and 10 *Forsythia* species using NGS technology. Our aims are: (1) to examine global patterns of structural variations in the cp genome and repetitive sequences of Forsythieae; (2) to reconstruct the phylogenetic relationship based on cp genome protein coding sequences and nuclear *cyc2*; and (3) to infer the biogeographic origin of *Forsythia* and explain their disjunctive distribution pattern.

## Materials and Methods

### Taxon Sampling

Three datasets were designed for this study. In the first phylogenetic analysis using cp genome data, we included 11 species—*Forsythia* (10 species) and *Abeliophyllum* (1 species)—to encompass the major lineages of Forsythieae based on the previous study by Kim and Kim (2011). We also included two cp genome sequences from other genera in Oleaceae (*Hesperelaea palmeri* A.Gray [GenBank no., LN515489] and *Olea europaea* L. [GenBank no., GU228899]) as outgroups. For the second phylogenetic analyses using *cyc2* sequences, in addition to 11 Forsythieae species (12 accessions), we included 14 species (21 accessions) from GenBank. To estimate the divergence time and biogeography, we analyzed Forsythieae species within a broad phylogenetic framework using the third dataset. This dataset included 72 species representing 25 genera of Oleaceae *sensu* Angiosperm Phylogeny Group [APG] (2016). The outgroup comprised 16 species from Verbenaceae (3 species), Byblidaceae (2), Carlemanniaceae (2), Plocospermataceae (1), Gelsemiaceae (1), Loganiaceae (1), Strychnaceae (2), and Rubiaceae (4) based on previous phylogenetic analyses (Wallander and Albert, 2000); their sequences were obtained from GenBank. The sampled taxa, localities, and voucher information are listed in Supplementary Table S1.

### DNA Sequencing, Assembly, Annotation, and Comparative Analysis

Total genomic DNA was extracted from silica-dried plant material using a DNeasy Plant Mini Kit (Qiagen, CA, United States). Genomic DNA was sequenced using an Illumina MiSeq2000 sequencer. The obtained raw reads were trimmed using Geneious v.7.1.9 (Kearse et al., 2012) to remove regions with chance of error greater than 0.05% per base. The cp genome sequences of 11 species were mapped on the reference genome of *O. europaea* using the ‘Map to Reference’ option implemented in Geneious. The mapping was conducted at medium-low sensitivity, and assembled reads were then *de novo* assembled with zero mismatches and gaps allowed among the reads. The reads were then re-aligned to the resulting *de novo* contigs with zero mismatches and gaps and with 100 iterations. Gaps and mismatches among the reads were not allowed in final assembled contigs. The cp genomes were annotated with DOGMA (Wyman et al., 2004) to identify coding sequences and rRNAs. The tRNA sequences were also confirmed by tRNAscan-SE (Lowe and Eddy, 1997). Genome maps were constructed using the Web-based tool GenomeVx (Conant and Wolfe, 2008). Visualization of the alignment of chloroplast sequences was conducted with mVISTA^1^, in which *O. europaea* was used as a reference sequence. Repetitive sequences were identified using REPuter program (Kurtz et al., 2001) and three types of repeats (forward, palindrome, and reverse) were identified following the procedure of Ni et al. (2016). The minimum repeat size was set to eight, and duplicated sequences in the IR region were excluded. We scored 1 (present) or 0 (absent) in a binary matrix for each minimum repeat size (Supplementary Table S2). Afterward, monomorphic bands across all species were discarded. To illustrate the genetic relationships at species levels, we analyzed the matrix of the minimum repeat sizes with the unweighted pair group method with arithmetic averages (UPGMA) based on pairwise distances (*p*-distance) using PAUP v.4.0 (Swofford, 2003).

The availability of some *cyc2* sequences in GenBank for *Forsythia a*nd *Abeliophyllum* allowed the comparison of a common marker from the nuclear genome. Row reads of the 11 Forsythieae species samples were assembled to *de novo* segments of *cyc2* region using default parameters in Geneious. We also checked the *cyc2* sequences from NGS by direct sequencing. The c*yc2* was amplified and sequenced using the primers Olea-CYC-126F and Olea-CYC693R as described in Zhong and Kellogg (2015).

### Phylogenetic Analyses

We derived phylogenies from the datasets of 78 coding genes of the cp genome and nuclear *cyc2* using maximum likelihood (ML) and Bayesian inference (BI) methods. Multiple-sequence alignment was performed in MAFFT v.6 (Katoh and Standley, 2013) using the default alignment parameters. Gaps were treated as missing data. The ML analysis was conducted on the RAxML BlackBox online server (Stamatakis, 2014), which supports GTR-based models of nucleotide substitution. The ML search option and the gamma model of rate heterogeneity were used to find the best scoring tree after bootstrapping. The statistical support for the branches (BS) was calculated by rapid bootstrap analyses with 1000 replicates (Stamatakis, 2014).

The BI analysis was conducted using MrBayes v.3.12 (Huelsenbeck and Ronquist, 2001; Ronquist et al., 2012). The best models of molecular evolution for the cp genome dataset (GTR + I + G) and *cyc2* (GTR + I) were evaluated in MrModeltest v.2.0 (Nylander, 2004). Four chains of Markov chain Monte Carlo (MCMC) were run simultaneously and sampled every 1000 generations for a total of 20 million generations. We plotted the log-likelihood scores of sample points against the generation time using Tracer v.1.5 (Rambaut and Drummond, 2014) to ensure that stationarity was achieved after the first 2,000,000 generations by checking whether the log-likelihood values of the sample points reached a stable equilibrium (Huelsenbeck and Ronquist, 2001). In addition, we used AWTY (Nylander et al., 2008) to compare split frequencies in different runs and to plot cumulative split frequencies to ensure that stationarity was reached. The first 5000 (25%) of sampled trees from each run were discarded as burn-in in Tracer v.1.5 (Rambaut and Drummond, 2014). The remaining trees were used to construct a 50% majority-rule consensus tree, and the proportion of trees that contained the clade was given as posterior probability (PP) on the consensus tree to estimate robustness of each clade.

### Estimates of Divergence Times

We used BEAST v.1.5.2^2^ (Drummond and Rambaut, 2007) to estimate divergence time based on the combined dataset of six cpDNA regions (*matK, rbcL, ndhF, atpB, rps16*, and *trnL-F*) (Supplementary Table S1). To generate input files for BEAST, the BEAUti interface was used in which a selected model (GTR + I + G) for the combined dataset was applied with a Yule speciation tree prior and an uncorrelated lognormal molecular clock model. Two runs of 100 million generations of MCMC chains were produced, sampling every 1000 generations. Convergence of the stationary distribution was checked by visual inspection of plotted posterior estimates in Tracer v.1.6 (Rambaut and Drummond, 2014). After discarding the first 10,000 trees as burn-in, the samples were summarized in the maximum clade credibility tree using TreeAnnotator v1.6.1 (Rambaut and Drummond, 2010) with a PP limit of 0.50 and summarizing mean node heights. Means and 95% higher posterior densities (HPDs) of age estimates are obtained from the combined outputs using Tracer. The results were visualized using FigTree v.1.3.1 (Rambaut and Drummond, 2012).

Calibrations of molecular phylogenetic trees are generally better when performed using multiple fossil records (Forest, 2009). Because there is no reliable fossil assigned to Forsythieae, we constrained the ages of four nodes from outgroups as follows: (C1) the crown age of *Olea* was constrained with a uniform distribution from 23 to 30 million years ago (mya) following Besnard et al. (2009) and Hong-Wa and Besnard (2013); (C2) the crown node of *Fraxinus* was constrained to 45 (±1.5) mya with a normal distribution based on the macrofossil of the genus described from the middle Eocene (Call and Dilcher, 1992); (C3) the crown age of *Byblis* was constrained to 45 (±1.5) mya with a normal distribution based on the fossil from the middle Eocene (Conran and Christophel, 2004); and (C4) the stem age of Oleaceae was constrained to 101.6 (±3.4) mya with a normal distribution following Roalson and Roberts (2016).

### Ancestral Area Reconstruction

The biogeographic data for the Forsythieae species were obtained from herbarium specimens and the literature (Chang et al., 1996). The distribution range of Forsythieae was divided into five regions: (A) Korea-Japan, (B) Central China, (C) East China, (D) Sikang-Yuennan, and (E) Western Europe following Takhtajan’s (1986) classification. The ancestral area reconstruction and the estimation of the spatial patterns of geographic diversification within Forsythieae were inferred using the Bayesian binary method (BBM) implemented in Reconstruct Ancestral State in Phylogenies (RASP) v.3.0 (Yu et al., 2015). The BBM was selected for its tendency to suggest single distribution areas for ancestral nodes compared to others and capability of providing precise and reliable results (Kim et al., 2015; Müller et al., 2015; Ito et al., 2017). For the biogeographic analysis, we used 100,000 trees obtained from the BEAST MCMC output after removing outgroups. The BBM was run with the fixed state frequencies model (Jukes–Cantor) with equal among-site rate variation for 50,000 generations, 10 chains each, and 2 parallel runs. The consensus tree used to map the ancestral distribution on each node was obtained with the Compute Condense option in RASP from stored trees. The maximum number of ancestral areas was set to five.

## Results

### Features of CpDNA Genomes in Forsythieae

**FIGURE 1:**
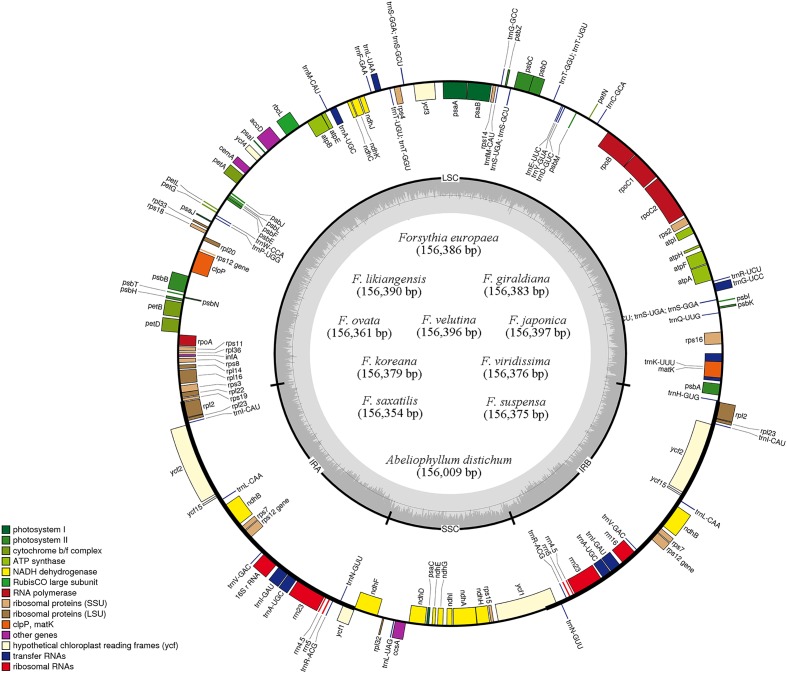
Gene maps of 11 Forsythieae chloroplast genomes. Genes drawn outside of the map circle are transcribed clockwise, while those drawn inside are transcribed counterclockwise. Genes belonging to different functional groups were color-coded. The darker gray in the inner circle corresponds to GC while the lighter gray corresponds to AT content.

The length of the complete cp genome sequences of 11 Forsythieae species ranged from 156,009 bp (*A. distichum*) to 156,397 bp (*F. japonica*) (**Figure 1** and **Table 1**). The mean genome coverages of the 11 cp sequences ranged from 80 (*F. velutina)* to 1830 (*F. giraldiana*). The GC contents of all Forsythieae species were identical (37.8%). Similar to other angiosperms, the Forsythieae cp genomes showed a typical quadripartite structure, consisting of a pair of IRs (25,682–25,711 bp) separated by the LSC (86,772–87,075 bp) and SSC (17,827–17,859 bp) regions (**Table 1**). The cp genomes were found to encode an identical set of 132 predicted functional genes, of which 114 genes were unique and 18 were duplicated in the IR regions. The 114 unique genes comprised 80 protein coding genes, 30 transfer RNA genes, and 4 ribosomal RNA genes (**Table 2**). The 18 duplicated genes in the IR region consisted of 7 coding genes (*rpl12, rpl23, rps7, rps12, ndhB, ycf2, ycf15*), 7 transfer RNA genes (*trnI-CAU, trnL-CAA, trnV-GAC, trnI-GAU, trnA-UGC, trnR-ACG, trnN-GUU*), and 4 ribosomal RNA genes (*rrn4.5, rrn5, rrn16, rrn23*) (**Table 2**). Fourteen genes, *rpl2, rpl16, rpoC1, trnA-UGC, trnI-GAU, trnK-UUU, trnL-CAA, trnL-UAA, trnV-UAC, ycf3, petB, petD, atpF, ndhA, ndhB*, and *clpP*, contained one intron, while two genes (*clpP, ycf3*) contained two introns. Partial *ycf1* was located at the boundary between IRa/SSC, leading to incomplete duplication of the gene within IRs. The *rps12* gene was *trans*-spliced, with the 5′ end of the transcript for *rps12* exon 1 were located in the LSC and the 3′ ends of exon 2 and exon 3 were duplicated in the IR regions. We found variation in indel events among Forsythieae species (**Table 3**); the smallest indel was 20 bp in length. The indels were specific and shared within the tribe Forsythieae. Specifically, a deletion of 416 bp in *accD*–*psaI* intergenic sequence (IGS) region was identified only in *A. distichum*, five indels were shared between *F. giraldiana* and *F. likiangensis*, and a deletion of 28 bp in *ndhF*–*rpl32* IGS region was observed in three species, *F. ovata*, *F. velutina*, and *F. japonica.*

**Table 1:** Summary of the chloroplast genome sequences used in this study.

Species	GenBank Accession	Total number of reads	Mean coverage	GC content (%)	Comparison of genome length (bp)			
					LSC	SSC	IR	Total
**Forsythieae**								
*A. distichum* Nakai	MF407183	12,995,214	542.8	37.8	86,772	17,827	25,704	156,009
*F. europaea* Degen & Bald	MF407184	22,561,724	1379.5	37.8	87,122	17,852	25,706	156,386
*F. giraldiana* Lingelsh.	MF407174	23,480,022	1830.0	37.8	87,176	17,843	25,682	156,383
*F. japonica* Makino	MF407175	12,634,318	260.5	37.8	87,131	17,844	25,711	156,397
*F. koreana* Nakai	MF407176	13,454,686	543.8	37.8	87,100	17,859	25,710	156,379
*F. likiangensis* Ching & Feng ex P.Y.Pai	MF407177	12,239,644	790.8	37.8	87,183	17,843	25,682	156,390
*F. ovata* Nakai	MF407178	9,732,160	486.7	37.8	87,095	17,844	25,711	156,361
*F. saxatilis* Nakai	MF407179	10,140,136	105.3	37.8	87,075	17,859	25,710	156,354
*F. suspensa* Vahl	MF407180	12,563,416	605.1	37.8	87,096	17,859	25,710	156,375
*F. velutina* Nakai	MF407181	11,480,024	80.1	37.8	87,130	17,844	25,711	156,396
*F. viridissima* Lindl.	MF407182	11,846,420	446.9	37.8	87,097	17,859	25,710	156,376
**Outgroups**								
*H. palmeri* A.Gray	LN515489	–	–	37.9	86,615	17,780	25,713	155,820
*O. europaea* L.	GU228899	–	–	37.9	86,614	17,791	25,742	155,889

**Table 2:** List of genes encoded in chloroplast genomes of Forsythieae.

Function	Gene group	Gene name
Self-replication	Large subunit of ribosome	*rpl*2^x2,Y 1^, *rpl*14, *rpl*16^Y 1^, *rpl*20, *rpl*22, *rpl*23^x2^, *rpl*32, *rpl*33, *rpl*36
	Small subunit of ribosome	*rps*2, *rps*3, *rps*4, *rps*7^x2^, *rps*8, *rps*11, *rps*12^x2,Y 1^, *rps*14, *rps*15, *rps*16, *rps*18, *rps*19
	Ribosomal RNA gene	*rrn*4.5^x2^, *rrn*5^x2^, *rrn*16^x2^, *rrn*23^x2^
	RNA polymerase subunits	*rpoA*, *rpoB*, *rpoC1*^Y 1^, *rpoC2*
	Transfer RNA genes	*trn*A-UGC^x2,Y 1^, *trn*C-GCA, *trn*D-GUC, *trn*E-UUC, *trn*F-GAA, *trnf*M-CAU, *trn*G-GCC, *trn*G-UCC, *trn*H-GUG, *trn*I-CAU^x2^, *trn*I-GAU^x2,Y 1^, *trn*K-UUU^Y 1^, *trn*L-CAA^x2,Y 1^, *trn*L-UAA^Y 1^, *trn*L-UAG, *trn*M-CAU, *trn*N-GUU^x2^, *trn*P-UGG, *trn*Q-UUG, *trn*R-ACG^x2^, *trn*R-UCU, *trn*S-GCU, *trn*S-GGA, *trn*S-UGA, *trn*T-GGU, *trn*T-UGU, *trn*V-GAC^x2^, *trn*V-UAC^Y 1^, *trn*W-CCA, *trn*Y-GUA
Photosynthesis	Photosystem I	*psa*A, *psa*B, *psa*C, *psa*I, *psa*J
	Photosystem II	*psb*A, *psb*B, *psb*C, *psb*D, *psb*E, *psb*F, *psb*H, *psb*I, *psb*J, *psb*K, *psb*L, *psb*M, *psb*N, *psb*T, *psb*Z
	Cytochrome	*pet*A, *pet*B^Y 1^, *pet*D^Y 1^, *pet*G, *pet*L, *pet*N
	ATP synthase	*atp*A, *atp*B, *atp*E, *atp*F^Y 1^, *atp*H, *atp*I
	Rubisco	*rbc*L
	NADH oxidoreductase	*ndh*A^Y 1^, *ndh*B^x2,Y 1^, *ndh*C, *ndh*D, *ndh*E, *ndh*F, *ndh*G, *ndh*H, *ndh*I, *ndh*J, *ndh*K
Other genes	Chloroplast envelope membrane protein	*cem*A
	ATP-dependent protease subunit P	*clp*P^Y2^
	Translational initiation factor	*inf*A
	Miscellaneous proteins	*acc*D*, ccs*A*, mat*K
Unknown function	Conserved reading frame	*ycf*1, *ycf*2^x2^, *ycf*3^Y 2^, *ycf*4, *ycf*15^x2^

*^x2^Two gene copies in IR; ^*Y1*^genes containing one intron; ^*Y2*^genes containing two introns*.

**Table 3:** Indels (Insertion/Deletion) identified in Forsythieae.

Loci	Species	Type of indel	Length (bp)	Sequence^a^
*rps16-psbK*	*A. distichum*	Deletion	29	AATTCATATTTCATATATAATTCATATAT
*rps4 - ndhJ*	*A. distichum*	Deletion	26	ATATATATTTATATATTTCGAATTCT
*accD – psaI*	*A. distichum*	Deletion	416	CAATTAGTTTATTTGTAGCAAACAAGTAGTTAGTTTATCAGAATCAAAGTAAATAAGAATGGAGTTTTC TTTGGTGACCTAAGATCTAATTGTAGAAATAATCAAAAGTTGCGGATAACTCTTTTTTTTTTACCTAGA ATCCCGATTACTAATTAAGATTAAGAAGTCTCTATCAACAAGATAAAAGAGTGAATTCTTCCTTTCGT GAAATTAGGCAAATAAAATAAAATGAATTTCGTCTTATGTATATAATCAAATAGAGAAAAGATAGATATA TAGTTTTTTATCTTTCTCTATCTCCCGAAAATCCCATTCTCGCTAAAAATTCCTGTTGGGTCGCATTC TAACGAATCTTTCGATAATCTGTAAGAAACTCTTTCTTTATTAAAAATTTGAAGACAAGAACAAAAGA
*psbM - psbD*	*F. giraldiana*	Insertion	23	AGAATAATTTCATTTCTAAAAAA
	*F. likiangensis*			
*petD - rpoA*	*F. giraldiana*	Deletion	20	GAAATAAAAGATTCAATTGG
	*F. likiangensis*			
	*A. distichum*			
*rps7 - ndhF*	*F. giraldiana*	Deletion	21	GTTAGTATTAGATTAGTATTA
	*F. likiangensis*			
	*F. europaea*			
	*A. distichum*			
*rps7 - ndhF*	*F. giraldiana*	Deletion	31	TCTTTGACAACACGAAAAACCATTGTTCAAC
	*F. likiangensis*			
*ndhF - rpl32*	*F. japonia*	Deletion	28	ATATTCTTCTTCTTTTTT(A**/**C**)**TATTTTTAG
	*F. ovata*			
	*F. velutina*			
	*A.distichum*			
*rpl32 - ccsA*	*F. giraldiana*	Deletion	29	AATGGATTTTTTTTGAGTTCTATCCTATT
	*F. likiangensis*			

^a^*Parentheses indicate the substitution among the species*.

**FIGURE 2:**
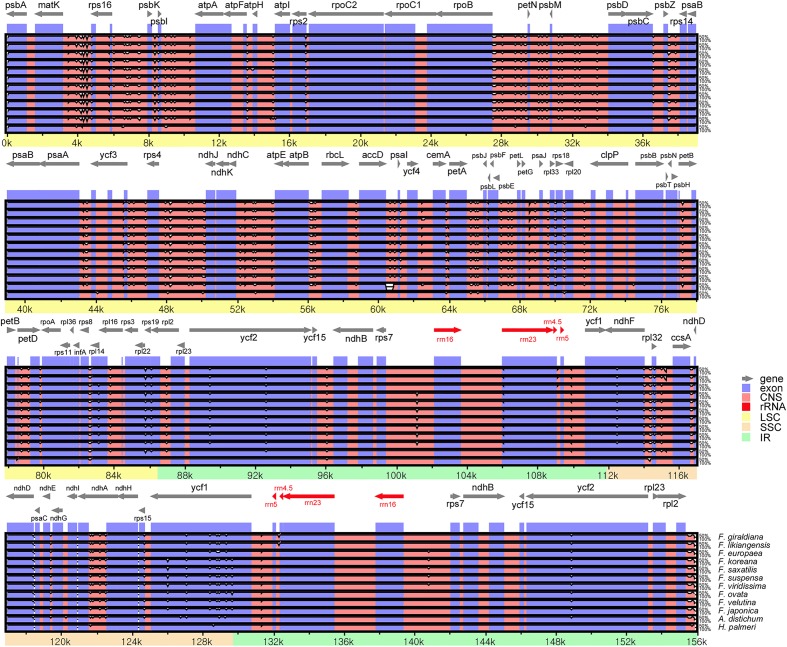
Sequence alignments of the Forsythieae chloroplast genomes using mVISTA program. VISTA-based identity plots showing sequence identity between 11 sequences of Forsythieae using *Olea europaea* (GenBank no., GU228899) as a reference. Black arrow shows the inverted repeats (IRs) in the chloroplast genomes.

Sequences were plotted to check their identity using the mVISTA tool by aligning the 11 Forsythieae cp genomes with the annotation of *O. europaea* as a reference (**Figure 2**). The whole aligned sequences indicate that the Forsythieae cp genomes are rather conservative although some divergent regions were formed between these genomes. Moreover, similar to other plant species, the coding region is more conservative than the non-coding counterpart.

### Repetitive Sequences

**FIGURE 3:**
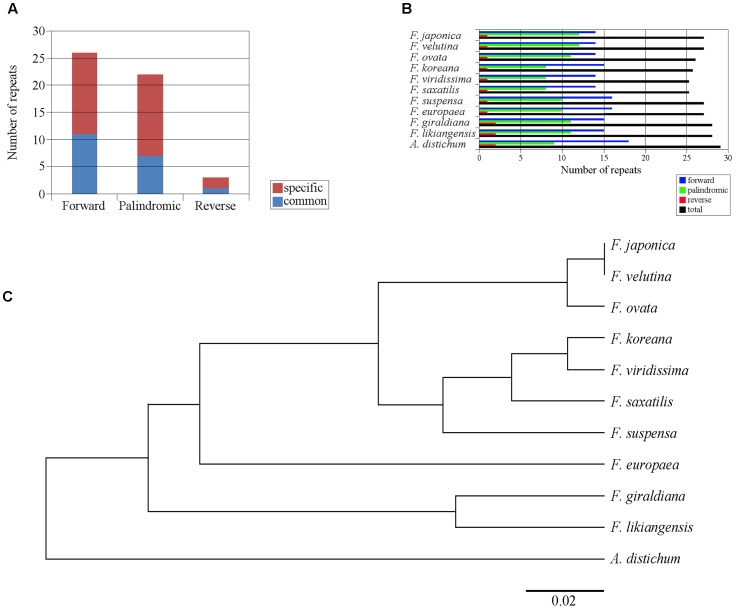
Repeat structure analyses in the 11 Forsythieae chloroplast genomes. **(A)** Histogram showing total number of the three repeat types in the Forsythieae. **(B)** Frequency of the three repeat types in each Forsythieae chloroplast genomes. **(C)** UPGMA tree based on the presence or absence of repeat structure.

Within the 11 cp genomes of the Forsythieae, we identified 51 repeat sequences, whose length varied from 17 to 41 bp. These repeat sequences comprised 26 forward (51%), 22 palindromic (41.1%), and 3 reverse (5.9%) sequences (**Figure 3A**). Among 11 Forsythieae species, the total number of repeats varied from 23 (*F. viridissima* and *F. saxatilis*) to 28 (*A. distichum*) (**Figure 3B**). Out of 51 repeats, 19 (37%) were commonly observed in Forsythieae and 32 were unique to one or more cp genomes (Supplementary Table S2). Eight repeats were specific to *A. distichum*. Similarly, a group of 13 repeats was shared by three species (*F. giraldiana*, *F. likiangensis*, and *F. europaea*). *F. ovata*, *F. velutina*, and *F. japonica* shared seven repeats; two of them can be used as potential markers for identifying these three species.

Using the dataset of repeat sequences, the UPGMA dendrogram of the 11 Forsythieae species revealed four defined groups: (1) *A. distichum*, (2) *F. likiangensis* and *F. giraldiana*, (3) *F. europaea*, and (4) the remaining species, subdivided into two clusters (a) *F. suspensa*, *F. saxatilis*, *F. viridissima*, and *F. koreana* and (b) *F. ovata*, *F. japonica*, and *F. velutina* (**Figure 3C**).

### Phylogenetic Analyses

**FIGURE 4:**
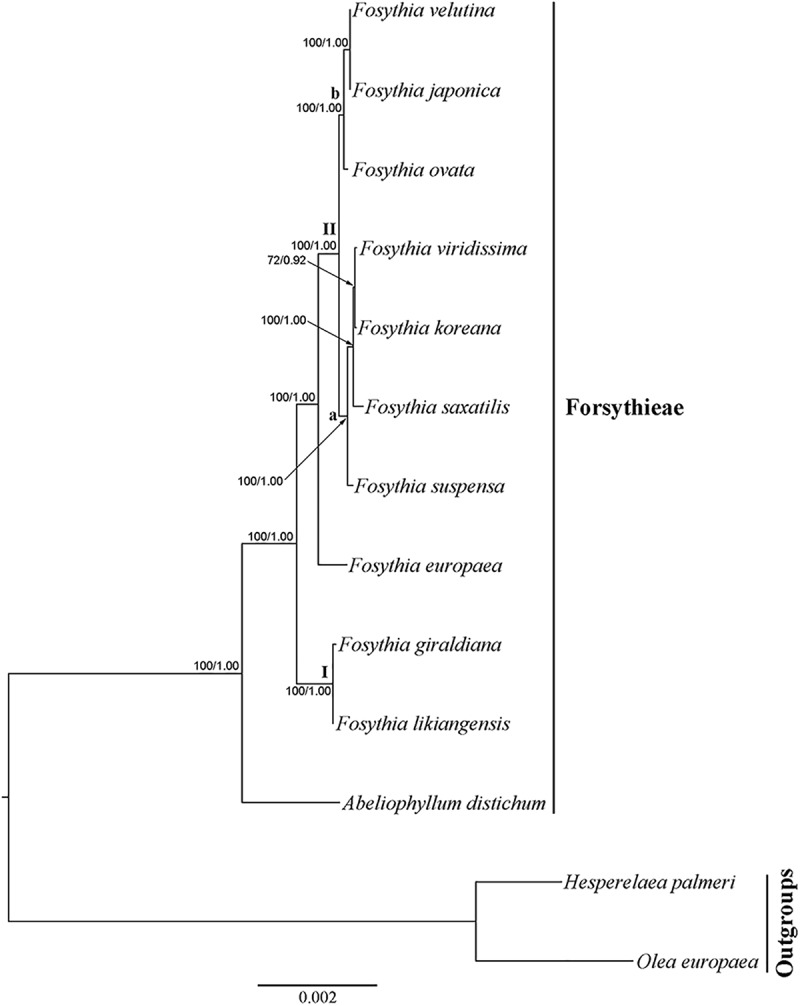
Phylogenetic tree resulting from Bayesian inference analysis of chloroplast 78 protein coding genes. Number above the branches indicate support values (maximum likelihood (BS)/Bayesian posterior probability (PP).

The cp genome dataset included 68,965 aligned nucleotides from 13 species and 78 protein coding genes, 1,684 (2.4%) of which were variable. The BI tree was identical in topology to the ML tree (**Figure 4**; ML tree not shown). All clades were strongly supported (BS = 100; PP = 1.00), except for *F. viridissima* and *F. koreana* (BS = 72; PP = 0.94). *Abeliophyllum* was sister to the *Forsythia* species. Two species (*F. likiangensis* and *F. giraldiana*; clade I) occupied the most basal position followed by the clade with *F. europaea*, which was sister to the rest of the Forsythieae species (clade II). The clade II species were divided into two subclades: (1) *F. suspensa*, *F. saxatilis*, *F. viridissima*, and *F. koreana* (subclade a) and (2) *F. ovata*, *F. velutina*, and *F. japonica* (subclade b).

**FIGURE 5:**
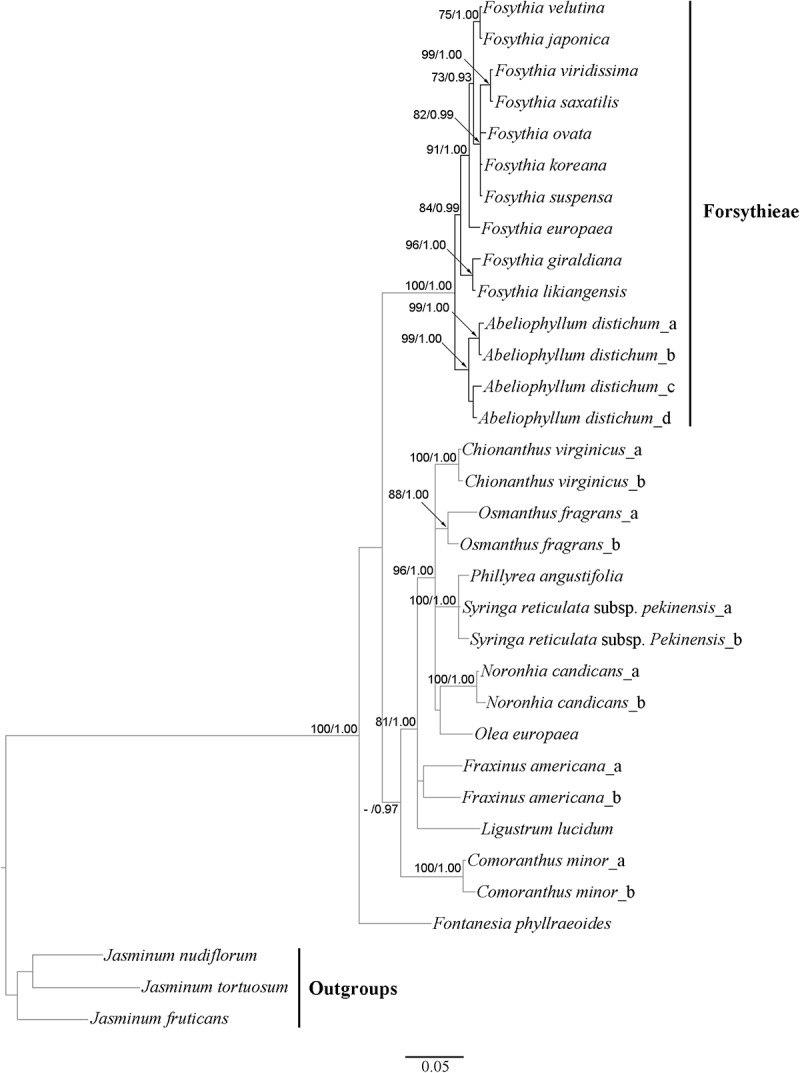
Phylogenetic tree resulting from Bayesian inference analysis of nuclear *cyc2* gene. Number above the branches indicate support values (maximum likelihood (BS)/Bayesian posterior probability (PP); a dash (–) indicates BP < 70%.

The aligned *cyc2* data matrix for 11 Forsythieae species (14 accessions) and 13 outgroup species (19) consisted of 582 nucleotides, 285 (50.0%) of which were variable. Within the ingroup, there were 40 (6.9%) variable sites. The ML tree (not shown) was identical in topology to the BI tree (**Figure 5**). Two conflicts were found in the positions of *F. ovata* and *F. viridissima* between cp genome and *cyc2* phylogenies. *F. ovata* was sister to *F. velutina*-*F. japonica* in the cp genome results (BS = 100, PP = 1.00; **Figure 4**), but was formed a clade with *F. viridissima–F. saxatilis–F. koreana–F. suspensa* in the *cyc2* phylogeny (moderately supported; BS = 82%, PP = 0.99; **Figure 5**). *F. viridissima* was sister to *F. koreana* in the cp genome tree (weakly supported; BS = 72, PP = 0.92), but was sister to *F. saxatilis* in the *cyc2* phylogeny (BS = 99, PP = 1.00). However, the topology of the *cyc2* tree is largely similar to that of cp genome tree in highly supported relationships.

### Divergence Times Analyses

**FIGURE 6:**
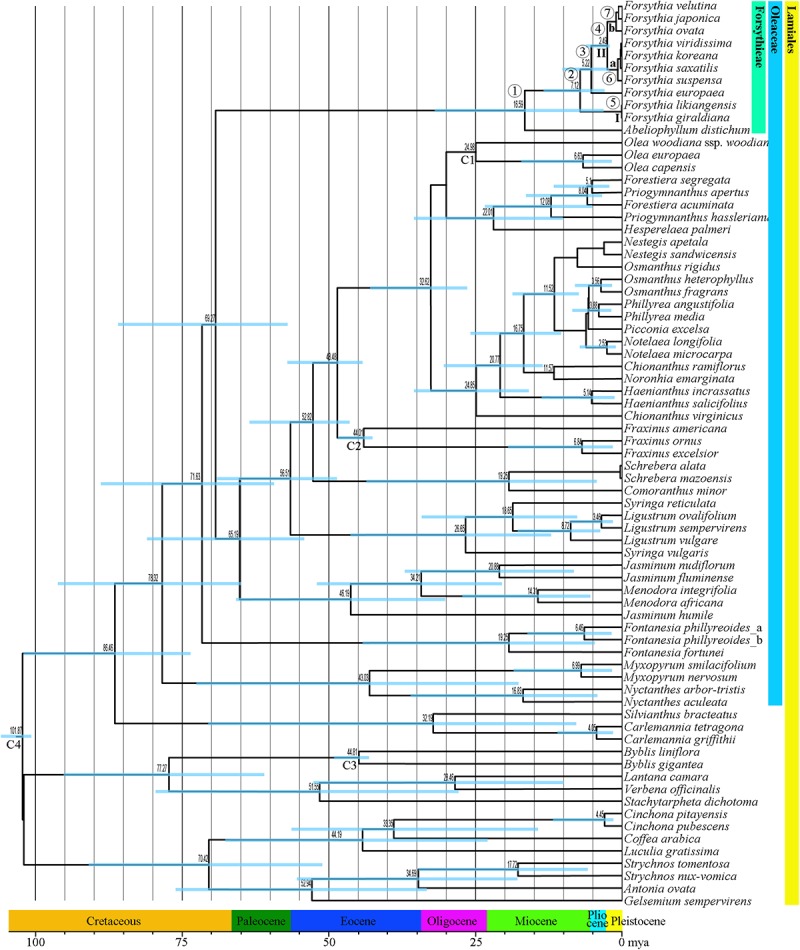
Chronogram showing divergence times estimated in BEAST based on six cpDNA sequence data (*matK, rbcL, ndhF, atpB, rps16*, and *trnL-F*). The divergence times are shown near each node. Blue bars represent 95% high posterior density for the estimated mean dates. The clades (I and II) and subclades (a and b) correspond to those in **Figure 4**. Nodes labeled C1–C4 are calibration points used in the analysis (for more details, see section “Materials and Methods”). Numbers 1–7 indicate nodes of interest (for details, see **Table 4**).

The mean divergence age estimates and 95% HPDs for nodes of interest based on the BEAST analysis of the combined dataset of six cpDNA sequences are presented in **Figure 6** and **Table 4**. The age of the crown node of Forsythieae was estimated at 16.6 mya (95% HPD = 5.0–33.6 mya; node 1) in the Miocene. The age of the most recent common ancestor of *Forsythia* was estimated at 7.1 mya (95% HPD = 2.6–12.9 mya; node 2) in the late Miocene. Within the genus, the age estimate of the crown node for *F. likiangensis* and *F. giraldiana* (clade I) was dated to be 0.3 mya (95% HPD = 1.2 × 10^-5^–1.2 mya; node 5) in the Pleistocene. The divergence time between Europe (*F. europaea*) and East Asia (*F. ovata*, *F. velutina*, *F. japonica*, *F. suspensa*, *F. saxatilis*, *F. viridissima*, and *F. koreana*; clade II) was estimated at 5.2 mya (95% HPD = 1.8–9.7 mya; node 3) in the late Miocene/Pliocene interface. The crown age of clade II was dated at 2.5 mya (95% HPD = 0.7–4.8 mya; node 4) in the Pliocene/Pleistocene interface. The age of the subclades was estimated to be the Pleistocene (subclade a, 0.9 mya, 95% HPD = 0.02–2.2 mya [node 6]; subclade b, 1.2 mya, 95% HPD = 0.2–2.5 mya [node 7]).

**Table 4:** Posterior age distributions of major nodes of Forsythieae with results of ancestral area reconstruction using BBM analysis.

Nodes^a^	Mean (95% HPD) (mya)	BBM (%)^b^
1	16.6 (5.0–33.6)	C (82) AC (17)
2	7.1 (2.6–12.9)	C (84)
3	5.2 (1.8–9.7)	C (80)
4	2.5 (0.7–4.8)	C (66), AC (25)
5	0.3 (1.2 × 10^-5^–1.2)	C (75) BC (11)
6	0.9 (0.02–2.2)	BC (41) C (35)
7	1.2 (0.18–2.4)	AC (87) C (12)

^a^*Node numbers and biogeographic codes correspond to those in **Figures 6**, **7**.*

^b^*Ancestral areas for each node are represented with ≥10%*.

### Ancestral Area Reconstruction

**FIGURE 7:**
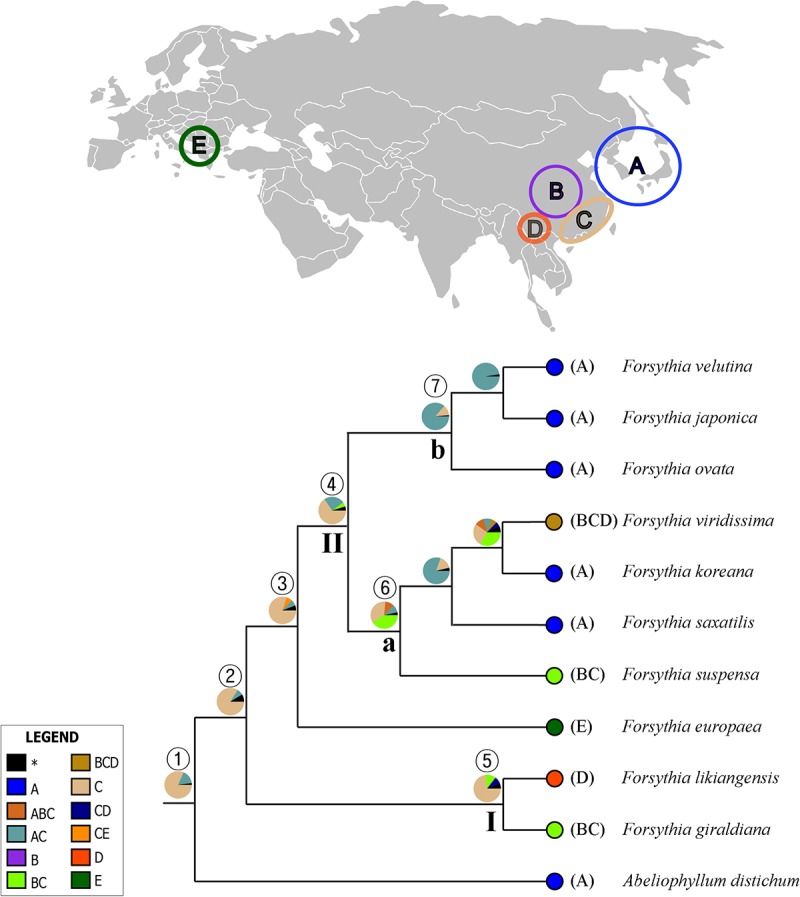
Summary of the Bayesian Binary Method (BBM) model of ancestral area reconstruction in Forsythieae based on a reduced BEAST combined-gene chronogram. The BBM ancestral area reconstructions with the highest likelihood are shown as pies for each clade of Forsythieae. Biogeographic regions used in BBM: A, Korea and Japan; B, Central China; C, East China; D, Sikang-Yuennan; E, West Europe. The clades (I and II) and subclades (a and b) correspond to those in **Figure 4**. Numbers 1–7 indicate nodes of interest (for details, see **Table 4**).

The summary of the ancestral ranges at the nodes of interest inferred by BBM are presented in **Figure 7** and **Table 4**. The BBM reconstruction suggests that Forsythieae (node 1) and *Forsythia* (node 2) originated in East China (C) with 82 and 84% marginal probabilities, respectively. Similar results were obtained for node 3 consisted of European (*F. europaea*) and East Asian *Forsythia* species, node 4 consisted of the clade II species, and node 5 which included SW Chinese *F. likiangensis* and Central and East Chinese *F. giraldiana* (clade I). The BBM analyses indicated two possible ranges, Central + East China (BC) and Korea and Japan + East China (AC), as the ancestral areas for the subclades (nodes 6 and 7); the occurrence of these ranges was 41 and 87%, respectively.

## Discussion

### Characteristics of Chloroplast Genomes in Forsythieae

In this study, we report the first complete cp genome sequences of 11 species from *Abeliophyllum* and *Forsythia* (Forsythieae) (**Figure 1** and **Table 1**). These cpDNAs exhibited a highly conserved pattern of gene content and order in comparison to the previously reported cp genomes of *O. europaea* and *H. palmeri* (Oleaceae). The LSC/IR and IR/SSC junctions were similar to those of a typical eudicot cp genome, but the length between junctions varied. Among the well-preserved junctions, *Forsythia* is divided into four groups. In other words, *Forsythia* species tend to have similar expansions/contractions within the group caused by intramolecular recombination between two short directly repeated sequences (Kim and Lee, 2004). Although the gene content and their order were highly conserved, we identified nine indels in the IGS region of the cp genomes of the 11 examined the Forsythieae species (**Table 3**). Repetitive sequences are patterns of DNA fragments that occur in multiple copies in the genome (Mehrotra and Goyal, 2014). They play a crucial role in higher plants due to their effectiveness in structural rearrangement and the expansion and contraction of chloroplast genome (SanMiguel et al., 1996). For example, the hairpin structure, created by the modification of the palindromic repeat sequence, affects the replication mechanism (Kurtz et al., 2001). Recently, the variation in repetitive sequences and SSRs has been used extensively in comparative genomic studies and species identification (Ni et al., 2016; Zhang et al., 2016). In the present study, the indels were not only unique, but they were also shared among the species of Forsythieae (**Figure 2**, **Table 3** and Supplementary Table S2), suggesting their value as molecular markers for species identification in the tribe. The UPGMA tree based on the presence/absence of the repetitive sequences recognized four clusters, which were congruent with the results of the phylogenetic analysis of the Forsythieae (**Figure 3C**).

### Phylogenetic Relationship within Forsythieae

Our phylogenetic analyses indicate that the sister group of *Abeliophyllum* with strong support (BS = 100%; **Figures 4**, **5**) is *Forsythia*. This result is consistent with previous studies of the molecular phylogenetic relationships of Oleaceae based on limited sampling of *Forsythia* (Kim, 1999; Lee et al., 2007; Kim and Kim, 2011). The sister relationship between *Abeliophyllum* and *Forsythia* is also supported by morphological and cytological evidence: (1) morphology of the corolla tube (Nakai, 1920), (2) unique form of polymorphism: heterostylous flowers (Ryu et al., 1976), and (3) same basic chromosomal number (*x* = 14; Taylor, 1945; Maekawa, 1962).

Our cp genome and nuclear phylogenetic analyses produced conflicting results regarding species relationships in *Forsythia* (**Figures 4**, **5**). Two striking differences between the two topologies are in the positions of *F. ovata* and *F. viridissima*. For example, in the cp genome tree, *F. ovata* is sister to *F. japonica*–*F. velutina* (BS = 100; **Figure 4**), the pattern also found by Kim and Kim (2011) using nrITS and two cpDNA regions. In contrast, the nuclear *cyc2* topology clustered *F. ovata*–(*F. viridissima*–*F. saxatilis*)–*F. suspensa*–*F. koreana* as a single moderately supported clade (BS = 82; **Figure 5**). Discordance between cp genome and nuclear phylogenies is common in plants (e.g., Soltis and Kuzoff, 1995; Deng et al., 2015). A possible explanation for the conflict has invoked introgression of cp genome from one species into the nuclear background of another (or *vice versa*) by interspecific hybridization, in which case the incongruent tree topologies represent the different histories of cp and nuclear genomes. Interspecific hybridization in *Forsythia* is not surprising by showing the heterostyly, with anther and stigma located at different height in the flowers (pin and thrum types; Rosati et al., 2007).

Within *Forsythia*, *F. giraldiana* and *F. likiangensis*, which are distributed in north-central and southern regions of China, respectively (Chang et al., 1996), formed a basal and strongly supported (BS = 100) clade (**Figure 4**). This relationship is also supported by similar morphological characteristics (ovate to elliptic leaf blade and entire leaf margin), and similar indels and repeat sequences in the cp genome analyses (Supplementary Table S2). This study is the first to conduct phylogenetic analysis of *F. likiangensis*, a species closely related to *F. giraldiana.*

In previous studies, the position of *F. europaea* within *Forsythia* was contentious. Based on RFLP and nuclear and plastid DNA sequence data, this species was placed as sister to *F. giraldiana* (Kim, 1999; Kim and Kim, 2011), whereas the RAPD analysis resolved it as sister to *F. viridissima* (Tae et al., 2005). Additionally, similar to *F. giraldiana* and *F. likiangensis*, *F. europaea* has oblong leaves with entire margins. In our analyses, *F. europaea* did not group with either *F. giraldiana* or *F. likiangensis*, but it was sister to the other two subclades comprised of seven *Forsythia* species (**Figure 4**). This point is reinforced by our *cyc2* phylogeny (**Figure 5**). This discrepancy between our and previous studies might have resulted from the addition of *F. likiangensis* in the data matrix and/or the use of the whole sequences of protein coding genes from the cp genome.

The remaining seven species, distributed in East Asia, were divided into two subclades in cp genome phylogeny (**Figure 4**). The first clade includes *F. ovata*, *F. japonica*, and *F. velutina*, and the monophyly of this group was supported by both morphological and molecular data (Lee, 1984; Kim and Kim, 2004; Lee, 2011). The second clade consists of *F. viridissima* (Southern China), *F. koreana* (Korea), *F. saxatilis* (Korea), and *F. suspensa* (widely distributed in China). In this clade, *F. suspensa*, the first described species in *Forsythia*, was sister to the remaining species; it is defined by apomorphic hollow stem and 3-parted to 3-foliolate leaf blade. However, the relationship of *F. viridissima*, *F. koreana*, and *F. saxatilis* presented herein did not corroborate the results of previous studies (Kim, 1999; Kim and Kim, 2011). *F. saxatilis* is characterized by ovate to lanceolate leaves and originally Nakai (1919a) treated it as variety *saxatilis* in *F. japonica.* Therefore, it is necessary to evaluate whether leaf shape is a key character in classifying the species within the genus *Forsythia* and conduct the taxonomic treatment of *F. saxatilis*.

### Historical Biogeography

Our molecular dating showed that the tribe Forsythieae originated in East China (**Figure 7**) and differentiated into two genera (*Forsythia* and *Abeliophyllum*) during the Miocene (16.6 mya, 95% HPD = 5.0–33.6 mya) (**Figure 6**). Due to the influence of the mid-Miocene climatic optimum (17–15 mya), the temperature in the Miocene was 4–5°C higher than it is today (You et al., 2009). Climate change offered an opportunity for accelerated establishment of the new lineage (Hinsinger et al., 2013). Therefore, Forsythieae were probably divided into different lineages during the period of climate change in the middle Miocene. The two genera of Forsythieae are clearly distinguished by their fruits (i.e., winged compressed indehiscent fruits in *Abeliophyllum* and dried capsule in *Forsythia*). The functional significance of samaras is to produce lift and drag to counter the forces of gravity, thereby reducing the falling speed of the fruit and increasing the distance, it may be dispersed by winds (Augspurger, 1986). Thus, the samara fruit may have been a key innovation for *Abeliophyllum*, triggering diversification and dispersal and conserved during its divergence and evolution.

Within *Forsythia*, *F. europaea*, a species restricted to areas in Europe (Green, 1972a; Willis and Shaw, 1973; Mabberley, 1997), originated in East China and along with its sister groups separated from the common ancestor at 5.2 mya (95% HPD = 1.8–9.7 mya) in the late Miocene–Pliocene interface (**Figure 6**). Based on low cpDNA substitution rate, Kim (1999) suggested relatively recent long-distance dispersal event at 0.46 mya (the Pleistocene) to explain the disjunction between Europe and East China. However, it is well known that age estimates based on a substitution rate from inter-species comparisons can be biased by heterogeneity in rates of molecular evolution (Pulquério and Nichols, 2006). This problem can be alleviated by analyzing multiple gene loci simultaneously and by using multiple calibration points (Yang and Yoder, 2003). In this study, we examined six cpDNA regions (*matK, rbcL, ndhF, atpB, rps16*, and *trnL-F*) across the broad sampling of Oleaceae and outgroups to address the divergence time and biogeographic origin of *Forsythia*.

Several factors, including the presence of the Turgai Strait, the Quaternary ice period, Qinghai-Tibetan Plateau (QTP) uplift, and climate change, in the Pliocene have been suggested as explanations for disjunct distribution of species between East Asia and Europe. In the Paleocene and early Oligocene, the Turgai Strait was a barrier that extended from the Arctic Ocean to the Tethys Seaway, separating the two regions (Legendre and Hartenberger, 1992); with its collapse the migration of the species was possible (Carlson et al., 2012). However, our divergence time between European and East Asian species is too recent to support this hypothesis (**Figure 6** and **Table 4**). Southern mountain chains stretching east to west between Asia and Europe provided possible paths for migration of plant species; however, the onset of the glacial period and the advancement of glaciers could have caused the extinction of species in the Pleistocene (Green, 1972b). This suggests that the Quaternary glaciers interrupted the migration route of *Forsythia*, isolating its populations into two areas, Europe and Asia. However, our results indicate that the two lineages split before the Pleistocene and therefore do not support this hypothesis. The uplift of the QTP in the Miocene prevented the dispersal of species between Europe and Asia (Harrison and Copeland, 1992; Axelrod et al., 1998; Sun et al., 2001; Zhang et al., 2006). In the case of *Forsythia*, the divergence time between European and East Asian species was estimated to 5.3 mya, thus supporting the hypothesis that vicariance due to the QTP uplift contributed to the disjunction in *Forsythia*. Finally, climate fluctuations during the Pliocene (Fiz-Palacios et al., 2010; Tu et al., 2010) probably influenced this disjunction as the rapid raise of the QTP resulted in drying and desertification of the regions around the QTP (Zhisheng et al., 2001; Xie et al., 2014). Similar to our results, climate change and glaciations were the main factors affecting the distribution of *Scabiosa* (Carlson et al., 2012). Thus, our results support the vicariance hypothesis and identify it as the main factor for the differentiation of *Forsythia* between Europe and Asia approximately 5.2 mya. The best hypothetical scenario to explain the distribution of *F. europaea* is the dispersal in the late-Miocene to Pliocene, followed by vicariance during the Pliocene climate fluctuations that caused the disjunction between European and East Asian populations. Seven species of *Forsythia* in East Asia (China, Korea, and Japan) diverged after the Pleistocene. However, it is difficult to interpret the distribution patterns of these species based on the current data and further phylogeographic studies at the population level should be carried out to understand the evolutionary history of these *Forsythia* species.

## Author Contributions

J-HK conceived and designed the experiments. Y-HH and KC collected the plant materials. Y-HH, CK, and J-HK performed the experiments and analyzed the data. Y-HH and CK wrote the draft and J-HK revised the draft. All authors agreed on the contents of the paper and declared that no competing interests exist.

## Conflict of Interest Statement

The authors declare that the research was conducted in the absence of any commercial or financial relationships that could be construed as a potential conflict of interest. The reviewer WW and handling Editor declared their shared affiliation.

## Footnotes

1 http://genome.lbl.gov/vista/index.shtml

2 http://beast.bio.ed.ac.uk
